# New Canine Medicines

**Published:** 1902-09

**Authors:** 


### NEW CANINE MEDICINES.

Bismuth Salicylas. This compound, obtained by treating nitrate of bismuth with a solution of sodium salicylate, is a white or nearly white powder, insoluble in water, alcohol, or glycerin, has been employed in human practice for some time in the various forms of diarrhœa and vomiting dependent upon gastric irritation, diarrhœa of typhoid, and of gastric intestinal catarrh. In canine practice it was first introduced by Mr. F. J. Taylor, M.R.C.V.S., Mosely, Birmingham, who employed it with considerable success in the epidemic of diarrhœa and vomiting in dogs supervening on attacks of influenza. From his clinical notes he found it invaluable in checking the relaxation and gastric vomiting, acting as an intestinal sedative and disinfectant to the alimentary tract. Mr. Taylor generally administers the drug in doses of 7 grains in small gelatin capsules, which are very easily given. His clinical reports of the drug have been confirmed by Mr. Frank Leigh, F.R.C.V.S., Bristol, and various other eminent canine practitioners. Some have administered it in the form of a mixture, suspended with mucilage or tragacanth.—J. Dowling Adams, in the *Veterinary Journal*, May, 1902.

Anthelmintics. *Capsules of Oil of Male Fern, with Turpentine.* These were first suggested to me some few years since by Mr. William Shipley, Jr., F.R.C.V.S., of Yarmouth, and are known among canine practitioners as "Shipley's Vermifuge Capsules," and, judging from the reports of numerous veterinarians, I don't know of any vermifuge that has given such entire satisfaction for the expulsion of intestinal worms (tæniæ of Lumbrici). Each capsule contains five minims of the oil of male fern and five minims of turpentine. Mr. John Cartwright, M.R.C.V.S., of Wolverhampton, has used these capsules in his extensive canine practice ever since their introduction, and has found them invaluable for the expelling of the different species of entozoa to which dogs are so prone. Like all anthelmintics, there are certain general rules which govern the administration of these capsules and which should not be lost sight of. Let the alimentary canal be as empty as possible, so that the combination of the drugs may act with the greatest force upon the entozoa. For this reason they are best administered in the morning and repeated for three or four mornings, and followed with a dose of castor oil.—*Ibid.*

